# Serine-associated one-carbon metabolic reprogramming: a new anti-cancer therapeutic strategy

**DOI:** 10.3389/fonc.2023.1184626

**Published:** 2023-08-18

**Authors:** Jing Zhang, Jian Bai, Chen Gong, Jianhua Wang, Yi Cheng, Jing Zhao, Huihua Xiong

**Affiliations:** ^1^ Department of Oncology, Tongji Hospital, Tongji Medical College, Huazhong University of Science and Technology, Wuhan, China; ^2^ Department of Obstetrics and Gynecology, Tongji Hospital, Tongji Medical College, Huazhong University of Science and Technology, Wuhan, China

**Keywords:** SERINE, one-carbon units, metabolism reprogramming, molecular targeted therapy, immunotherapy

## Abstract

Tumour metabolism is a major focus of cancer research, and metabolic reprogramming is an important feature of malignant tumours. Serine is an important non-essential amino acid, which is a main resource of one-carbon units in tumours. Cancer cells proliferate more than normal cells and require more serine for proliferation. The cancer-related genes that are involved in serine metabolism also show changes corresponding to metabolic alterations. Here, we reviewed the serine-associated one-carbon metabolism and its potential as a target for anti-tumour therapeutic strategies.

## 1 Introduction

Although cancer genomics is the central topic in tumour research, the correlation between tumour and metabolism needs to be evaluated in detail. One of the hallmark events during tumorigenesis is the evolution of metabolic patterns that sustain tumour proliferation and survival (1). Metabolic reprogramming is an essential feature of malignant tumours to provide enough energy and essential bioactive substances for tumour growth (2). Therefore, some scholars have proposed that tumour is not only a genetic disease but also a metabolic disease (3). This hypothesis originated from the discovery of the Warburg effect or aerobic glycolysis (4). Subsequently, various anti-metabolic drugs have been used in anti-tumour therapy.

One-carbon metabolism refers to the process in which some amino acids form a group containing a carbon atom (also called a one-carbon unit), which participates in the biosynthesis after being transferred. One-carbon unit cannot exist in the free form and is bound to the N5 and N10 positions of tetrahydrofolate (THF), which is a key coenzyme that undergoes transformations to enable the transfer and participation of one-carbon units in metabolism (5). Serine is a non-essential amino acid, which is the third major contributor to cancer cell metabolism after glucose and glutamine. The amino acid participates in the formation of nucleic acids, lipids, amino acids and coenzymes and is also required for cell proliferation (6). The one-carbon units derived from serine contribute to the synthesis of purines and thymidine, methylation reactions, and the production of NADPH involved in anti-oxidant defences in cells (5).

Here, we reviewed the role of serine-associated one-carbon metabolism in different cancers and addressed the possibility of targeting this metabolic pathway as an anti-tumour therapeutic strategy.

## 2 Serine and one-carbon metabolism

The intracellular one-carbon units are mainly derived from the catabolism of amino acids, such as glycine, histidine, serine, tryptophan, and methionine. These units are produced by pathways involved in serine/glycine metabolism, glycine cleavage system, and other amino acid metabolism (4). Serine is the major contributor of one-carbon units in cancer metabolism. On the one hand, cancer cells can take up exogenous serine quickly; on the other hand, cancer cells can synthesise serine (7). Consequently, cancer cells ensure optimal serine supply and enough one-carbon units to meet their requirements.

In recent years, over-activated serine/glycine metabolic pathways have been implicated in the development of cancer (8). The serine *de novo* synthesis pathway becomes the main source of serine in the absence of serine or during rapid cell proliferation. Cancer cells obtain most of their energy needs through aerobic glycolysis, and the anabolism of serine is an important branch of glycolysis (9). The process consists of three enzymatic reactions. First, 3-phosphoglycerate (the intermediate metabolite of glycolysis) is converted to 3-phosphohydroxypyruvate (pPYR) by 3-phosphoglycerate dehydrogenase (PHGDH) with the simultaneous conversion of oxidised nicotinamide adenine dinucleotide (NAD+) to reduced nicotinamide adenine dinucleotide (NADH) (10). Next, phosphoserine aminotransferase 1 (PSAT1) catalyses the conversion of pPYR to phosphoserine (pSER) and α-ketoglutarate. Finally, pSER is dephosphorylated by phosphoserine phosphorylase (PSPH) to form serine (11). Serine is converted to glycine by serine hydroxymethyltransferase (SHMT)1 and SHMT2 in the cytoplasm and mitochondria, respectively. SHMT transfers methyl group from serine to THF and participates in the folic acid cycle (12). SHMT2 catalyses the production of the one-carbon unit from serine in the rapidly proliferating cells, and this unit enters the cytoplasm in the form of formic acid to participate in nucleotide synthesis. In SHMT2-deficient cell lines, SHMT1 catalyses serine catabolism to fulfil the requirements of one-carbon groups for thymidine and pyrimidine synthesis (13).

## 3 Metabolic reprogramming of serine in cancers

Cancer cells require more serine because of their higher proliferative capacity than normal cells. Moreover, their genes associated with serine metabolism also show changes corresponding to metabolic changes (7).

PHGDH is a key enzyme required at the first step of the serine *de novo* synthesis pathway. Possemato (14) reported that the PHGDH gene is in a genomic region of recurrent copy number gain in breast cancer, and the PHGDH protein levels are elevated in 70% of estrogen receptor-negative breast cancers. Breast cancer cells with a high PHGDH expression have increased serine synthesis flux. Song et al. suggested that the expression of PHGDH is increased in pancreatic cancer tissues compared with the adjacent normal tissues. Increased expression of PHGDH is associated with tumour size, lymph node metastasis, and TNM stage of patients with pancreatic cancer. Therefore, PHGDH could serve as an important prognostic indicator and therapeutic target for pancreatic cancer (15).

PSAT1 is a key enzyme in serine biosynthesis, and its overexpression is associated with colon, non-small cell lung, and breast cancers (16). Liao et al. found that high PSAT1 expression is associated with a poor prognosis of nasopharyngeal cancer and is an indicator of an advanced cancer stage, indicating that PSAT1 is a potential prognostic biomarker (16). PSAT1 is an independent prognostic factor for colorectal cancer. It is overexpressed in the colorectal cancer tissues compared with the normal tissues, and its overexpression is associated with a response to chemotherapy using irinotecan, 5-fluorouracil, and leucovorin and shorter survival time (17).

PSPH is a key enzyme catalysing the last step of the serine synthesis pathway. Sun (9) found that aberrant expression of PSPH is highly correlated with mortality in patients with hepatocellular carcinoma. Li (18) described that most colorectal cancer cell lines overexpress PSPH, which supports cancer growth during 5-FU treatment. Therefore, PSPH can be targeted for increasing the anti-cancer efficacy of conventional therapy in patients with colorectal cancer.

SHMT is an important enzyme in the one-carbon unit cycle associated with serine. It also participates in the rapid proliferation of various cancers; therefore, SHMT is an actively studied target for anti-cancer drug development. Sun (19) reported that stromal SHMT1 expression was highest in anaplastic carcinomas, tumoural SHMT1 was higher in poorly differentiated carcinomas and papillary thyroid carcinomas, and stromal SHMT1 expression was associated with short disease-free survival in follicular variant papillary thyroid carcinomas. Dual SHMT1/2 knockout blocked HCT-116 colon cancer xenograft formation, suggesting that both SHMT1 and 2 may be involved in the association between serine metabolic reprogramming and lymphoma. However, the role of SHMT2 in mitochondria may be more crucial (12).

## 4 Serine deprivation for cancer therapy

Approximately 40%–80% of patients with cancer suffer from malnutrition. Cancer cells can obtain energy to support their survival and growth even in nutrient-deficient conditions (20). Both exogenous and endogenous serine support the development of cancer (2, 7, 21, 22). Cancer cells rely on exogenous serine to maintain their high proliferation rate. Therefore, serine deprivation has anti-cancer effects in patients with different genetic backgrounds and cancer types.

p53 mutations make colon cancer cells sensitive to serine deprivation because they cannot induce the necessary G1 blockade in vivo. The cells lacking p53 cannot respond to serine depletion, leading to oxidative stress. Such cells cannot guide the residual serine to produce glutathione for nucleotide synthesis. Additionally, these mutations can result in decreased viability and severely impaired cancer cell proliferation, indicating that serine deprivation may have a potential role in the treatment of p53-deficient cancers (23).

Serine deprivation can also effectively inhibit spontaneous cancer growth driven by Apc loss (intestinal cancer) or Myc activation (lymphoma) (21). Pancreatic ductal adenocarcinoma (PDAC) mice on the serine-free diet showed a 50% reduction in cancer growth. The researchers also used an FDA-approved drug LOXO-101 to prevent axons from entering PDAC. The drug blocked the activation of the receptor protein on the surface of neurons that interacted with nerve growth factor (also known as TRK-A), thereby inhibiting cancer innervation. The use of this drug alone could not slow down the growth of PDAC cancers in mice. However, a combination of this drug with the serine-free diet reduced the growth rate of PDAC by 50% compared with the diet alone. This suggests that nerves are necessary to support the growth of PDAC cells in serine-deprived cancer regions. TRK inhibitors have been approved for the treatment of TRK fusion cancers, and approximately 40% of patients with PDAC who cannot produce serine after surgery may be treated with a combination of TRK inhibitors and a low-serine diet to reduce cancer recurrence. However, this strategy has not been tested in clinical trials (24).

Serine deprivation can also enhance the effect of cancer therapy through the mitochondrial complex I inhibitor metformin (or phenformin). However, in vivo experiments showed that neither phenformin alone nor serine deprivation could inhibit the growth of allogeneic colon adenocarcinoma in mice, but their combination produced a marked inhibitory effect (25). Naama Kanarek concluded that a serine-free diet is a promising approach to inhibit cancer progression, which should be tested in combination with other cancer treatment methods in patients with diverse genetic backgrounds. However, experiments conducted with cell lines showed that serine depletion alone was more effective than simultaneous deprivation of serine and glycine. Therefore, future research should consider serine deprivation alone, rather than the combination of serine and glycine deprivation (26). Therefore, serine (but not glycine) deprivation supports the reduction of one-carbon metabolism, which then inhibits cancer cell proliferation.

## 5 Development of serine synthesis pathway inhibitors for cancer SSP therapy

The serine synthesis pathway is catalysed by several enzymes, such as PHGDH, SHMT1/2, and MTHFD1/2, and their suppression may decrease cancer cell growth and survival. However, drugs targeting these enzymes are still in the initial stages of development, and the identification of appropriate inhibitors will be crucial for cancer treatment (27).

### 5.1 Inhibitors targeting PHGDH

PHGDH catalyses the first rate-limiting step of the serine biosynthesis pathway and is highly expressed in certain cancers, such as triple-negative breast cancers (28), melanoma (28), cervical cancer (11), and gliomas (29). PHGDH amplification is associated with oncogenesis, and PHGDH knockdown or silencing shows anti-cancer effects in vivo and in vitro. Therefore, PHGDH is a promising cancer therapeutic target, and several PHGDH inhibitors have been identified as anti-cancer molecules (30). These molecules inhibit the proliferation of PHGDH-dependent cancer cells, suggesting the potential role of serine biosynthesis in one-carbon unit metabolism (31).

Pacold et al. (2) tested two potent PHGDH inhibitors, NCT-502 and NCT-503, (**Table 1**) in cancer cell lines and transplanted cancers. These inhibitors showed effective anti-cancer activity against PHGDH-dependent cancers but not against PHGDH-non-dependent cancers. PHGDH inhibitors did not disturb the synthesis of other amino acids, except serine and aspartic acid. Moreover, PHGDH inhibitors not only affected the synthesis of serine from glucose, but also reduced the incorporation of intracellular and extracellular serine into the nucleotide of the one-carbon unit. However, some questions are still unanswered, and future research should focus on the mechanism of action of PHGDH inhibitors and the most appropriate time of their use during the course of the disease (22).

**Table 1:** Drugs targeting serine-related one-carbon metabolic pathway.

Target	Drugs	Indications	Status
PHGDH	NCT-502	triple-negative breast cancer, melanoma cell lines, cervical cancer, and gliomas	In development
	NCT-503		In development
SHMT1/2	AM-807/42004511	lymphoblastic leukaemia	In development
	AM-807/40675298		In development
	AM-807/42004633		In development
	AGF347	lung adenocarcinoma, colon cancer, and pancreatic adenocarcinoma	In development
MTHFD 1/2	LY345899	Triple-negative breast cancer	In development
	DS18561882	Acute myeloid leukaemia	In development

Ngo et al. (32) found that cancer cells colonising the brain face a microenvironment lacking exogenous serine and glycine. Increased serine synthesis is important for the production of nucleotides and subsequent proliferation of highly invasive brain metastatic cells. PHGDH suppression and drug-induced inhibition of serine synthesis attenuated brain metastasis and improved the overall survival in mice but did not inhibit the growth of extracranial cancers. These results indicated that the extracellular amino acid-restricted environment in the brain enhanced the susceptibility of metastatic cancer to PHGDH inhibitors and serine synthesis inhibition. Overall, this study provided a theoretical basis for the use of PHGDH inhibitors to treat brain metastases (32).

Targeted inhibition of PHGDH may affect cancer growth only in serine-poor environments. Serine content is low in the cancer microenvironment (33). Specific PHGDH inhibitors that do not affect cancer growth in the standard medium may still be effective in slowing cancer growth in vivo, in part because of their systemic rather than local anti-cancer activity. Some small molecule inhibitors targeting PHGDH in PHGDH-dependent cancer cells have been successfully developed and verified in vitro. These molecules not only inhibit cancer cell proliferation, but also reduce the growth of xenograft cells (2, 27, 34).

### 5.2 Inhibitors targeting SHMT1/2

Cancer cells require SHMT1/2 for optimal tumorigenicity and proliferation, illustrating the importance of serine metabolism in cancers. Some anti-folates, such as pemetrexed and methotrexate, can bind to and inhibit human SHMT in vitro and have been approved by FDA at high concentrations (≥100 μM) (35–37). Moreover, small compounds inhibiting SHMT1/2 have been identified for cancer treatment.

Pandey et al. (38) found that the knockdown of SHMT1 by targeted small interfering RNAs (siRNAs) reduced the tumour size in the xenograft model of mice. The compound “2.12” had anti-cancer activity in the mid-micromolar range with selective activity against SHMT1 (12). The cysteine reactive inhibitor 3-bromopyruvate was selective for SHMT1 because of the absence of cysteine residues at the active site of SHMT2 (39).

The number of currently available SHMT1-selective inhibitors is 2–3 times higher than that of SHMT2 inhibitors (12). However, breakthroughs have been achieved with the discovery of SHMT2-targeting compounds in vivo (40). Nonaka et al. (41) directly measured SHMT activity using a novel fluorescence method and screened more than 200k compounds. They got two “hits” with sub-micromolar activity; “Hit 1” was selective for SHMT1, whereas “Hit 2” was the first SHMT2 selective inhibitor. Knockdown of SHMT2 in colorectal cancer xenografts completely blocked cancer development only when SHMT1 was also downregulated. Han et al. established a direct screening system for the SHMT2 inhibitors and found three compounds with non-competitive and medium-strength binding to SHMT2, namely AM-807/42004511, AM-807/40675298, and AM-807/42004633 (**Table 1**) (42).

SHMT1 and SHMT2 have high sequence homology and many inhibitors target them simultaneously. Several dual SHMT1/2 inhibitors were optimised, including SHIN1 and SHIN2. SHIN1 had poor stability and a short half-life in vivo. SHIN2 did not show the pharmacologic defects of SHIN1 and demonstrated synergistic therapeutic effects with methotrexate in T-cell acute lymphoblastic leukaemia xenografts (12, 40, 43).

AGF347—the first in vivo active SHMT inhibitor—is a folate mimetic showing a broad-spectrum anti-cancer effect (**Table 1**). The molecule showed an inhibitory effect in lung adenocarcinoma, colon cancer, and pancreatic adenocarcinoma cell lines (44, 45), and a notable efficacy was also observed in vivo in pancreatic adenocarcinoma models (44). Therefore, AGF347 is a potent inhibitor of SHMT1/2.

Targeting both SHMT1 and SHMT2 is essential for SHIN1/2, AGF347 and other compounds to exert potent anti-cancer effects. Simultaneous targeting prevents the metabolic reaction due to the loss of SHMT2 activity by SHMT1 reversal and the metabolism of glycine and 5, 10-methylene THF (12). However, this hypothesis needs to be verified in vitro and in vivo.

### 5.3 Inhibitors targeting MTHFD1/2

MTHFD is highly expressed in several cancers and is related to overall survival. Patients with high MTHFD levels have a poor prognosis; however, the specific function and mechanism of MTHFD are still unclear. Therefore, exploring new therapeutic targets for MTHFD may contribute to the treatment of cancers.

MTHFD1L is a metabolic enzyme that regulates formate production in the folate cycle (46). Cui et al. (47) confirmed MTHFD1L as the downstream target of melatonin, which was markedly upregulated in head and neck squamous cell carcinoma (HNSCC). MTHFD1L overexpression was related to the poor prognosis of patients with HNSCC. Moreover, MTHFD1L promoted the progression of HNSCC both in vivo and in vitro and reversed the anti-cancer effect of exogenous melatonin. Importantly, formate could partially rescue malignant phenotypes that were inhibited by Mthfd1l-knockout or exogenous melatonin. In addition, melatonin suppressed the expression of MTHFD1L in HNSCC cells and tissues by downregulating the phosphorylation of cyclic AMP-responsive element-binding protein 1 (CREB1). These findings revealed novel melatonin–pCREB1–MTHFD1L–formate regulatory axis in HNSCC. Taken together, MTHFD1L–formate axis promoted the progress of HNSCC, and melatonin inhibited HNSCC development through CREB1-mediated MTHFD1L and formate downregulation.

MTHFD2 is one of the most differentially expressed metabolic enzymes in cancers and is involved in DNA replication and genomic stability of cancer cells (22, 48). Although some MTHFD2 inhibitors were reported, only two compounds, LY345899 and DS18561882, (**Table 1**) showed anti-cancer activity in vitro and in vivo (43, 49). LY345899 was the first described MTHFD2 inhibitor. The IC50 values of LY345899 for inhibiting MTHFD1 and MTHFD2 were 6396 nM and 535 nM, respectively, indicating its weak inhibitory activity against MTHFD2 and more selectivity for MTHFD1 (2, 50). The first truly selective inhibitor of MTHFD2, DS44960156, was reported in 2019, although its potency was insufficient (51). DS18451882 was 90-fold more selective for MTHFD2 than for MTHFD1 and was effective against MDA-MB 231 triple-negative breast cancer xenografts in immunocompromised mice in vivo (52). Therefore, DS18561882 is a promising inhibitor targeting MTHFD2.

Helleday et al. screened a small molecule inhibitor of MTHFD2. This inhibitor reduced the replication fork velocity and induced replication stress in acute myeloid leukaemia cells in vitro and in vivo followed by cell cycle arrest at the S-phase and apoptosis. Mechanistically, MTHFD2 inhibitors blocked thymidine production, led to the misincorporation of uracil into DNA, and induced replication stress. These results demonstrated the functional relationship between MTHFD2-dependent cancer metabolism and replication stress, which can be therapeutically manipulated with this novel inhibitor (52).

## 6 Seine metabolism and immunotherapy

Cancer immunotherapy has made substantial progress in the treatment of various cancers over the past decade. A core phenomenon of malignancies is metabolic dysfunction, which reprograms metabolic homeostasis in cancers and stromal cells and affects metabolic modifications of specific proteins. These post-translational modifications, such as glycosylation and palmitoylation, alter the localisation, stability, and function of proteins. Most of these proteins are involved in the development and progression of cancers, as well as in acute or chronic inflammation. Therefore, metabolic modifications targeting immune checkpoints and inflammation offer an attractive therapeutic strategy for certain cancers (53). Palmitoylation is a process that covalently binds palmitic acid to protein residues in three different ways, namely S-palmitoylation, O-palmitoylation and N-palmitoylation. O-palmitoylation is linked to serine/threonine residues by oxygen ester bonds. In 2019, two research groups reported that S-palmitoylation maintained the stability of PD-L1 and suppressed T-cell cytotoxicity (54). However, additional research is required to explore the influence of serine and O-palmitoylation on PD-L1 in different cancers.

Ma et al. revealed that serine is an essential metabolite for the optimal expansion of T cells after antigen stimulation. Restriction of dietary serine damages the pathogen to drive the expansion of T cells in vivo, but does not affect the overall immune cell homeostasis. Mechanistically, serine provides glycine and one-carbon units for *de novo* nucleotide biosynthesis in proliferating T cells, and one-carbon units from formate can rescue T cells from serine depletion. The authors suggested that serine—as a critical immune metabolite —directly regulated adaptive immunity and the responsiveness and tolerance of cancer immunotherapy by controlling the proliferation of T cells (55).

Tregs are also affected by acid metabolism. Serine promotes the synthesis of glutathione and enters a carbon metabolic network, the key process of the Teff reaction. The specific deletion of the catalytic subunit of glutamate–cysteine ligase (Gclc) from mouse Tregs resulted in the loss of Treg-specific glutathione (56). Kurniawan et al. (56) found that glutathione-deficient Tregs showed increased serine metabolism and decreased FoxP3 expression. Additionally, Treg-specific Gclc-deficient mice showed an enhanced anti-cancer response. When mice were fed a serine-deficient diet to suppress serine supply, Gclc-deficient Tregs restored FoxP3 expression and immunosuppressive ability of mice (57).

Phosphatidylserine (PS) is mainly localised on the inner side of the phospholipid bilayer of the cell membrane in healthy cells. It migrates to the outer lobe during apoptosis. Wang et al. established PSout cancer models, in which cancer cells lacked the CDC50A component of PS turnover enzyme and continued to expose PS but still survived. The everted PS restricted the expression of MHC-I/II in tumour-associated macrophages, thereby inhibiting cancer antigen presentation and promoting the development of cancer. The PS receptor Tim-3 (but not Tim-4) mediates the recognition of PS in cancers. The research team also established PSin models when cancer cells lacked the PS-disrupting enzyme Xkr8 in vivo and cannot expose PS during normal apoptosis. The inversion of PS results in the accumulation of apoptotic cells and activates cGAS to produce cGAMP, thereby activating the type I interferon signalling pathway of immune cells in the cancer microenvironment. These immune cells, including tumour-associated macrophages and natural killer cells, work together to inhibit cancer growth. Simultaneously, the concentration of IL-10, a cytokine that suppresses anti-cancer immunity, was also reduced in the PSin models. Moreover, Xkr8 inactivation in combination with the anti-PD-1 regimen resulted in the complete elimination of cancers in mice. Although no small molecular drugs targeting Xkr8 are currently available, the silencing of Xkr8 in vivo with short hairpin RNA or siRNA resulted in externally blocked PS and powerful therapeutic oncostatic effects (58). Therefore, PS in cell membrane, which is synthesised from serine, may be targeted in cancer immunotherapy.

## 7 Conclusion

Serine-related one-carbon metabolism is important for the occurrence and development of cancers. Currently, researchers are actively exploring this metabolic pathway to develop clinically effective anti-tumour drugs, which can be used independently or in combination with immunotherapy. However, most of these drugs are in the research and development stage and have not yet entered the clinical trials. Therefore, their efficacy and safety in the real world need to be further confirmed.

## Author contributions

JZhang wrote the ‘Introduction’, ‘Development of serine synthetic pathway’, and ‘Inhibitors for cancer therapy’ sections. JB wrote the ‘Serine deprivation for cancer therapy’ section. JZhao and HX designed the review and revised the manuscript. CG wrote the ‘Serine and one-carbon metabolism’ section. JW wrote the ‘Metabolic reprogramming of serine in cancers’ section. YC wrote the ‘Serine deprivation for cancer therapy’ section. All the authors approved the manuscript.
